# Mucinous cystic neoplasms of the mesentery: a case report and review of the literature

**DOI:** 10.1186/1477-7819-7-47

**Published:** 2009-05-19

**Authors:** Georgios Metaxas, Athanasios Tangalos, Polyxeni Pappa, Irene Papageorgiou

**Affiliations:** 1 University Hospital of South Manchester, The Nightingale and Genesis Prevention Centre, Southmoor Road, M239LT, Manchester UK; 2 Helena Venizelos General Hospital, 2nd Department of Surgery, GR-11521, Athens, Greece; 3 Drama General Hospital, Department of Surgery, Drama, Greece

## Abstract

**Background:**

Mucinous cystic neoplasms arise in the ovary and various extra-ovarian sites. While their pathogenesis remains conjectural, their similarities suggest a common pathway of development. There have been rare reports involving the mesentery as a primary tumour site.

**Case presentation:**

A cystic mass of uncertain origin was demonstrated radiologically in a 22 year old female with chronic abdominal pain. At laparotomy, the mass was fixed within the colonic mesentery. Histology demonstrated a benign mucinous cystadenoma.

**Methods and results:**

We review the literature on mucinous cystic neoplasms of the mesentery and report on the pathogenesis, biologic behavior, diagnosis and treatment of similar extra-ovarian tumors. We propose an updated classification of mesenteric cysts and cystic tumors.

**Conclusion:**

Mucinous cystic neoplasms of the mesentery present almost exclusively in women and must be considered in the differential diagnosis of mesenteric tumors. Only full histological examination of a mucinous cystic neoplasm can exclude a borderline or malignant component. An updated classification of mesenteric cysts and cystic tumors is proposed.

## Background

Cysts of the mesentery, retroperitoneum and omentum present with similar incidence in both sexes, varying between 1:260,000 and 1:27,000 in adults and 1:20,000 in children. They are usually incidental, or present with unspecific and chronic symptoms involving abdominal pain, distention, a palpable mass, gastrointestinal and urinary obstruction [1-3]. Acute manifestation is more often described in children and infants and may be associated with rupture [4-8], hemorrhage [9], torsion [10], infection or complicated hernia [11]. A 3% malignancy rate has been demonstrated [1].

### Mucinous cystic neoplasms

Mucinous cystic neoplasms (MCNs) arise in the ovary and various extra-ovarian sites, predominantly but not exclusively [1,12-16] in adult females. The similarities between ovarian [17] and extra-ovarian MCNs suggest a common pathway of development. The cyst wall of extra-ovarian MCNs [18] is lined by mucin-secreting flat, cuboidal and/or columnar epithelium associated with an underlying subepithelial ovarian like stroma (OLS). OLS is documented by histological features (spindle shaped cells and myofibroblastic proliferation on electron microscope study) and immunohistochemistry (positivity for vimentin, *α*-smooth muscle actin and desmin) [19-22]. Although the presence of OLS is considered a requisite diagnostic criterion for MCNs, this is not always identified. MCNs have been extensively described in the pancreas [18-27], the appendix [28-30] and the hepatobiliary tract [31,32] and more rarely in the retroperitoneum [33-35] paratesticular tissues [36-41], lung [42-44]breast [45-47], spleen [18,48,49] bowel [50] and the mesentery.

## Case presentation

**Figure 1:**
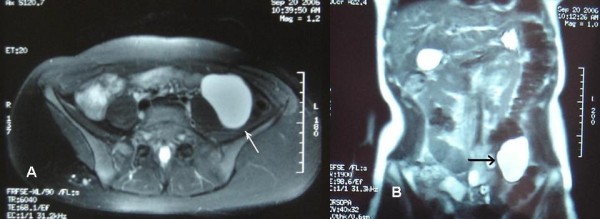
**(a, b): MR scan appearance of the cystic tumour (arrow)**.

**Figure 2:**
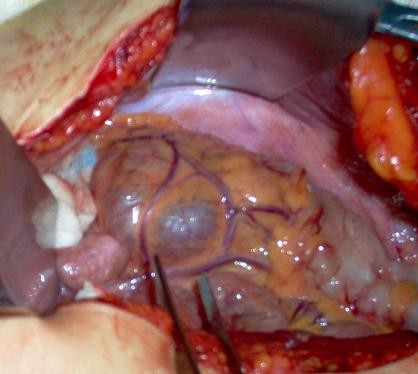
**Intra-operative appearance, medial view of the mesentery, inferion mesenteric vessels lying on the cyst surface**.

**Figure 3:**
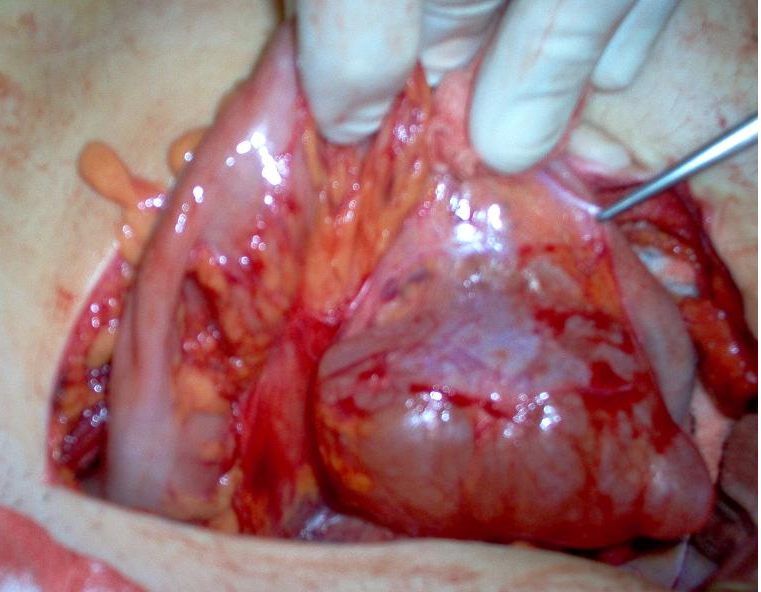
**Lateral view of mesentery, cyst enucleation in an avascular plane**.

**Figure 4:**
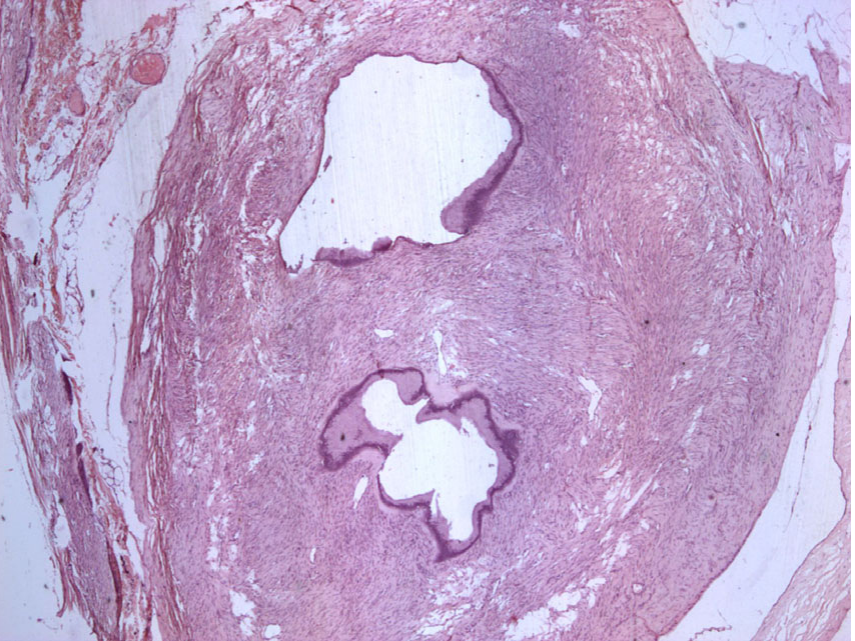
**Microscopic appearance of the cyst wall, ovarian like stroma, epithelial lining**.

**Figure 5:**
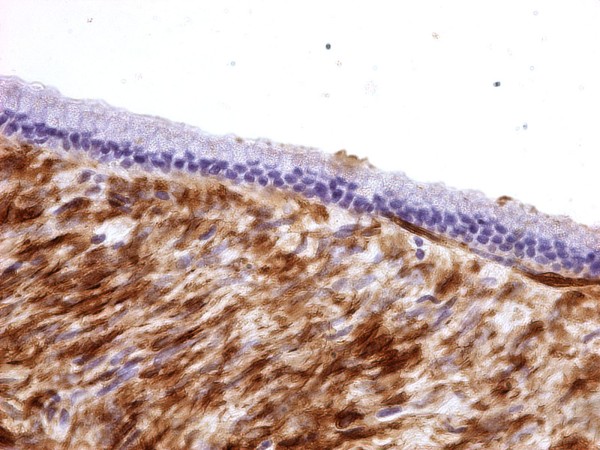
**Benign columnar mucinous epithelium lining of the cyst wall**. Immunohistochemistry reveals stromal positivity for actin.

**Figure 6:**
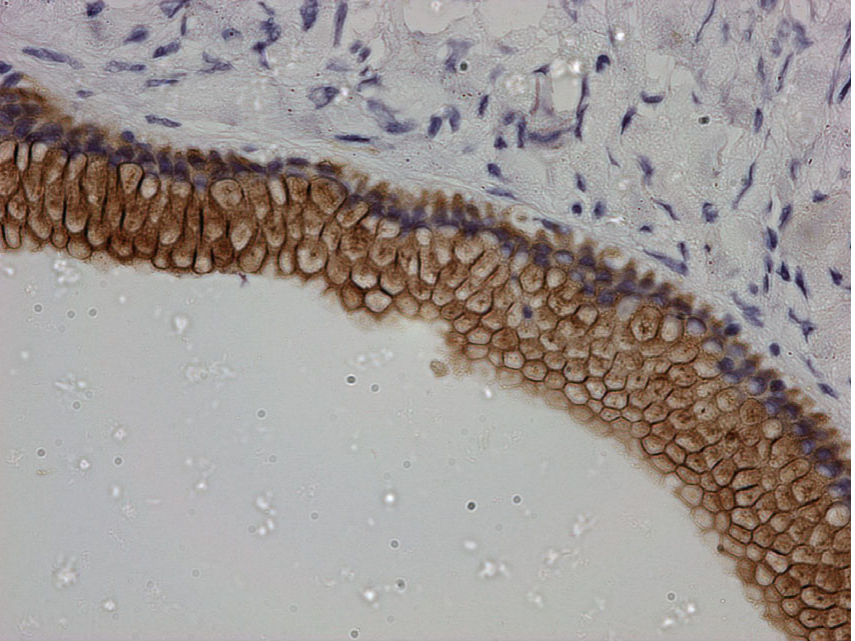
**Immunohistochemistry, epithelial positivity for CK 7**.

**Figure 7:**
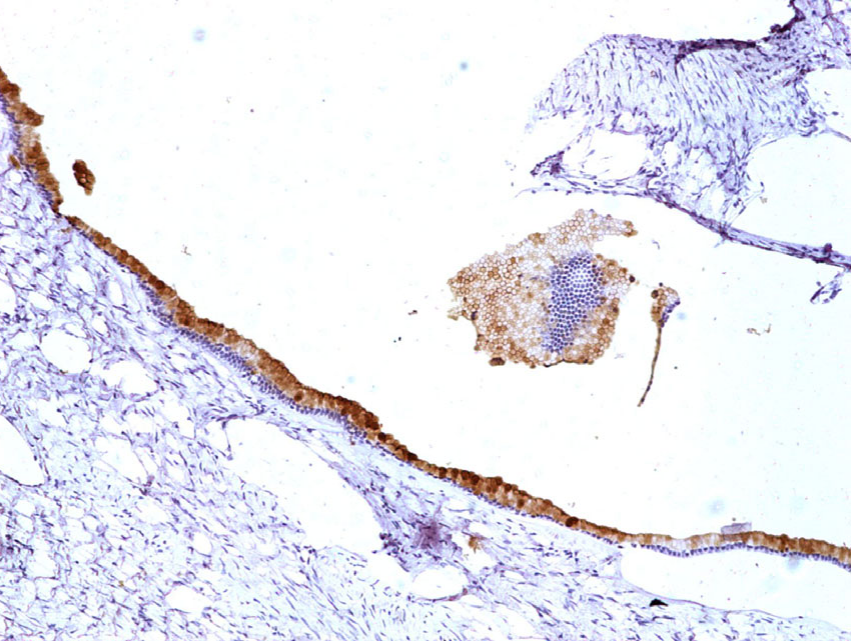
**Immunohistochemistry, epithelial positivity for CA-125**.

A 22 year old white-Caucasian female, with otherwise unremarkable history, presented with chronic, left sided, vague abdominal pain. There were no abnormal findings on clinical examination. Ultrasound (US), computerized tomography (CT) and magnetic resonance (MR) scans (Fig. 1a, b) demonstrated a unilocular cystic mass measuring 8.5 × 6 × 3.5 cm and lying medially to the descending colon. No definite preoperative diagnosis could be established. At laparotomy the mass was fixed within the descending and sigmoid colonic mesentery (Fig. 2). As there were no firm adhesions or shared blood supply (Fig. 3), enucleation was easily performed. The cyst had a macroscopically thin and smooth wall and contained white-yellowish fluid. The cyst wall was examined in its entirety. Histology demonstrated two distinct components: an outer ovarian-like stromal layer, composed of densely packed spindle-shaped cells (Fig. 4) and an inner epithelial layer, consisting of cuboidal and columnar mucinous cells (Fig. 5, 6). Immunohistochemical study of the stromal cells demonstrated positivity for vimentin, actin, and desmin. The epithelial cells showed positivity for cytokeratin-7 (Fig. 6), CA-125 (Fig. 7), CEA, and CA 19-9 and negative expression of cytokeratin-20. There was no cellular atypia. The overall features suggested a benign neoplasm of epithelial origin with the appearance of an ovarian mucinous cystadenoma. The patient recovered uneventfully and remained well on annual follow-up with abdominal US.

## Discussion

There are thirteen documented cases of mesenteric MCNs in the literature prior to this report (Table 1). Five of those originated from the mesentery of the small intestine [15,18,51-53], one from the mesoappendix [54] and seven from the mesocolon [55-60]. The only male patient was a five year old child with an unresectable neoplasm [15]. Clinical presentation typically involved chronic abdominal pain (n = 8) and distention (n = 5). Three patients were asymptomatic and one presented with acute manifestation of symptoms. Interestingly, preoperative imaging was inconclusive in nine cases, suggested ovarian origin in four cases, and mesenteric origin in only one case. No pathognomonic malignant features were illustrated. Pathology reported nine benign mucinous cystadenomas (including the present case), three borderline MCNs, (based on the presence of atypical nuclei, pseudo-stratification, glandular arrangements and lack of invasion) and two carcinomas. The median age at diagnosis of benign mesenteric MCNs was 26.1 yrs, compared to 35.2 yrs for non-benign tumours. One of the carcinomas [51] was thought to be the result of malignant transformation following earlier incomplete excisions of a recurrent benign tumour. One of the patients with a borderline tumour, presented with metastatic disease in the mediastinal lymph nodes four years after removal, which may represent a missed invasive focus due to incomplete examination of the entire neoplasm wall. This patient had initially undergone a partial colectomy and salpingo-oophoretomy [55]. Another two patients underwent partial colectomies due to cyst wall adhesions, while enucleation of the tumor was performed in six. Partial cystectomy was performed in two unresectable tumors. Immunohistochemistry was reported in four cases. There was no report of ectopic ovarian tissue or evidence of teratomatous developement. There was no association between the age and tumor size.

**Table 1:** Reported cases of mesenteric mucinous cystic neoplasms of the large intestine (1–8), small intestine (9–13) and appendix (14). *No details contained in published article.

s	Reference	Age Sex	Clinical presentation	Imaging tests – correlated diagnosis	Size (cm)	Site	Operation	Histology – Immunohistochemistry
1.	Banerjee et al. (1988) [55]	58 F	Incidental finding	US, Uncertain	7	Hepatic flexure	Right Hemicoletomy	Benign mucinous cystadenoma
2.		38 F	Pain, distention	US, Uncertain	11	Descending colon	Colectomy Salpingo-oophorectomy	Borderline malignant MCN
3.	McEvoy et al. (1997) [56]	24 F	Pain, constipation distension.	US, Ovarian origin	20 × 15	Sigmoid colon.	Enucleation	Benign mucinous cystadenoma (CAM 5.2, CEA)+, Factor VIII -
4.	Linden et al. (2000) [58]	32 F	Incidental finding	US, CT, Uncertain	13 × 10 × 10	Transverse colon	Enuleation	Mucinous cystadenocarcinoma
5.	Vrettos et al. (2000) [59]	38 F	Pain, nausea, vomiting, distention, oedema of the lower limbs	US, CT Mesenteric cyst	17 × 12	Sigmoid colon.	Enucleation	Borderline malignant MCN
6.	Talwar et al. (2004) [57]	32 F	Acute pain, vomiting, urinary frequency, constipation	US, Ovarian origin	10 × 7 × 5	Descending colon	Left hemicolectomy	Borderline malignant MCN
7.	Swaveling et al. (2008) [60]	18 F	Asymptomatic abdominal swelling	US, CT, Uncertain Adjacent to R kidney	15	Right hemicolon	Enucleation	Benign mucinous cystadenoma
8.	Present case	22 F	Pain	US, CT, MRI, Uncertain	8.5 × 6 × 3.5	Descending + sigmoid colon	Enucleation	Benign mucinous cystadenoma (CK7, CEA, CA19-9, CA125, actin, desmin, vimentin)+, ck20(-),
9.	Cohen et al (1988) [52]	36 F	Found during pregncy	Uncertain	40	Ileum	Cyst resection	Benign mucinous cystadenoma
10.	Bury and Pricolo (1994) [51]	36 F	Pain, reccurent unresectable cysts én a 13 year period,	CT	*	Small intestine Unspecified	Partial cyst resections	Incomplete excision, transformation to carcinoma CK+, EMA, CEA, B72.3, Leu M1
11.	Czubalski et al. (2004) [53]	38 F	N/A	Ovarian origin	N/A	N/A	N/A	Benign mucinous cystadenoma
12.	Shioho et al (2006) [18]	14 F	*	*	15	*	*	Benign mucinous cystadenoma
13.	Luo et al (2008) [15]	5 M	Abdominal pain and swelling	None	20–25	Small intestine Unspecified	Unresectable, Segmental excision	Benign mucinous cystadenoma, (biopsy only) ER, PR (-), Inhibin (+), OLS +
14.	Felemban & Tulandi (2000) [54]	20 F	Abdominal pain, back ache	US, Ovarian origin	7.6 × 7 × 5.3	Appendix	Lap. Enucleation, appendicectomy	Benign mucinous cystadenoma

### Pathogenesis and biologic behavior of extra-ovarian MCNs

The origin of extra-ovarian MCNs has been sporadically attributed to implanted or ectopic ovarian tissue [61], supernumerary ovaries [62,63] or mono-phyletic developement of a teratoma component [64-66]. A recent concept linked the development of hepatic and pancreatic MCNs to the migration of epithelial cells from the embryonic gonads during early foetal life [67]. The most widely accepted theories for the pathogenesis of extra-ovarian MCNs include: Coelomic metaplasia of epithelial cells or invaginated peritoneum along the course of ovarian descent, mucinous metaplasia in pre-existing mesothelial cysts and neoplastic differentiation of epithelial cells from a secondary extragenital Mullerian system [1,2,51,68-71].

According to the WHO classification (ICD 10), MCNs are divided into benign adenomas, borderline tumours, non-invasive (in situ) and invasive carcinomas. The malignant potential of all MCNs is supported by observations of malignant transformation of benign neoplasms during long term follow up [24,51]. Other authors noticed a frequent concurrence of benign and focally borderline or/and malignant epithelium [21,72,73]. Also, as illustrated in the present study and previous pancreatic MCNs series [72,74], the median age at diagnosis is higher for malignant MCNs, which implies progression from adenoma to carcinoma. Consequently, failure to excise or study the entire cyst wall may result in the miscategorization of a neoplasm [75].

When differentiating mucinous from non mucinous neoplasms and non neoplastic cysts and evaluating their malignant potential, the following features may have a positive predictive but not pathognomonic value: Patient age and tumour size [27,74], multilocularity, presence of calcifications [58], intracystic papillary projections or mural nodules [20], presence or lack of OLS [20,76], nuclei atypia, co-expression of (a6)-integrin and p53 immunoactivity [20,77], high viscosity and/or high levels of CEA in the cyst fluid and positivity of other tumours markers (Ca 19-9, Ca-125, Ca 15-3) [24,78,79]. Oestrogen and progesterone receptor positivity in stromal cells varies [80].

### Classification of mesenteric cysts and cystic tumors

None of the recognized classifications of mesenteric cysts [81-85], has included MCNs up to date. Malignant mesothelioma is suggested as the only potential primary cystic malignancy in the mesentery. We found three more cases of primary mesenteric cystic neoplasms in the literature [86-88], as well as reports of mesenteric hydatid [8,89-92]] and tuberculous cysts [93-95]. In view of the existing data, we propose an updated classification of mesenteric cysts and cystic tumors, as shown in the Appendix.

### Diagnosis and treatment

There is no definitive diagnostic test. Radiological examinations may suggest the origin of a cyst, but cannot exclude the malignant potential of an MCN [35,82,84,96-99]. Guided aspiration cytology and fluid analysis is rarely diagnostic, has a high false negative incidence [100] and may only be helpful in the complicated management of pancreatic cystic masses [101,102]. The definite diagnosis remains postoperative and therefore intra-operative frozen section does not have any use. Following exclusion of non-surgical conditions, complete excision is the only treatment option for all mesenteric neoplasms [103-105] because of their malignant potential as well as the high recurrence rate of benign tumors – such as lymphangiomas and mesotheliomas – after incomplete resection [106-108]. Open or laparoscopic approach depends on the surgeon's skills, as more complicated resections may be required [109-111]. Only one mesenteric MCN has been treated laparoscopically up to date, by a gynaecologist [54].

## Conclusion

Mucinous cystic neoplasms of the mesentery present almost exclusively in women and must be considered in the differential diagnosis of mesenteric tumors. Whilst there are no pathognomonic diagnostic criteria, a mesenteric cyst should be approached as potentially malignant especially in adults. Only complete excision and full histological examination of a mucinous cystic neoplasm can exclude a borderline or malignant component. We propose an updated classification of mesenteric cysts.

## Consent

Written informed consent was obtained from the patient for publication of this case report and any accompanying images. A copy of the written consent is available for review by the Editor-in-Chief of this journal

## Competing interests

The authors declare that they have no competing interests.

## Authors' contributions

GM and AT were involved in the treatment of the patient, study design and drafting of the manuscript. GM also reviewed literature, prepared the tables and figures and edited the final version. PP and IP collected case details and helped in the literature search and drafting of the manuscript. All authors read and approved the final manuscript.

## Appendix

### Proposed updated classification of mesenteric cysts and cystic tumors

#### Mesenteric Cysts and Cystic Tumors

Cysts of lymphatic origin

• Simple lymphatic cyst

• Lymphangioma

Cysts of mesothelial origin

• Simple mesothelial cyst

• Benign cystic mesothelioma

• Malignant cystic mesothelioma

Cysts of enteric origin

• Enteric duplication cyst

• Enteric cyst

Mucinous cystic neoplasms

• Mucinous cystadenoma

• Borderline malignant mucinous cystic neoplasm

• Mucinous cystadenocarcinoma

Cysts of urogenital origin

Miscellaneous neoplasms

• Mature cystic teratoma

• Neuroendocrine carcinoma

• Cystic spindle cell tumor

Non – neoplastic cysts

• Hydatid cyst

• Tuberculous cyst

Non – pancreatic pseudocysts

• Haematoma

• Abscess
